# Unveiling the Metabolic Burden: A Clinicopathological Study of Chronic Plaque Psoriasis and Its Association With Metabolic Syndrome

**DOI:** 10.7759/cureus.89289

**Published:** 2025-08-03

**Authors:** Siddharth Shah, Kunjal Parikh, Monal L Shah, Atul Kothari

**Affiliations:** 1 Department of Pathology, Sumandeep Vidyapeeth University, Vadodara, IND; 2 Department of Pathology, Thadiq General Hospital, Thadiq, SAU; 3 Department of Pathology, Mahatma Gandhi Memorial Medical College, Indore, IND

**Keywords:** chronic plaque psoriasis, dyslipidemia, histopathology, metabolic syndrome, psoriasis comorbidities

## Abstract

Introduction

Psoriasis is a chronic, immune-mediated inflammatory skin disease with systemic manifestations. Among its significant comorbidities, metabolic syndrome (MS) - a constellation of obesity, hypertension, dyslipidemia, and insulin resistance - has gained recognition due to its association with increased cardiovascular risk and reduced life expectancy. Chronic systemic inflammation, shared immunological pathways, and elevated pro-inflammatory cytokines are thought to underlie this association. This study aimed to explore the relationship between histopathologically confirmed chronic plaque psoriasis and MS.

Methods

A single-center, cross-sectional, hospital-based observational study was conducted between January 2022 and June 2023 at Smt. B. K. Shah Medical Institute and Research Centre, Gujarat. A total of 43 patients with histopathologically confirmed chronic plaque psoriasis were enrolled. Skin biopsies were stained with hematoxylin-eosin and analyzed microscopically. Metabolic parameters, including lipid profile, fasting blood glucose, and HbA1c, were measured using automated analyzers. MS was diagnosed based on National Cholesterol Education Program Adult Treatment Panel III criteria. Patients were categorized into two groups: MS (n=6, 14%) and non-MS (n=37, 86%). Clinical severity was assessed using the Psoriasis Area and Severity Index (PASI) and Body Surface Area (BSA) scoring systems. Statistical analysis was performed using IBM SPSS Statistics (IBM Corp., Armonk, USA), with significance set at p<0.05.

Results

Six out of 43 patients had MS (14%), with a mean age of 47.83 ± 21.78 years and male predominance of 83.3%. MS was more prevalent among married (6/6, 100%), middle-class (4/6, 66.7%), and non-vegetarian (5/6, 83.3%) male patients. PASI scores were higher (5-6) in 4/6 (66.7%) patients within the MS group, though this difference was not statistically significant. However, moderate BSA involvement (3-10%) was significantly more common among MS patients than non-MS patients. Specifically, 4/6 MS patients (66.7%) had moderate BSA involvement, whereas the majority of non-MS patients had mild involvement (<3%). This difference was found to be statistically significant (p=0.036). Compared to non-MS patients, those with MS had significantly higher weight, BMI, waist circumference, and systolic and diastolic blood pressure (all p<0.05). Biochemically, MS patients exhibited significantly elevated levels of random blood glucose, total cholesterol, triglycerides, very low-density lipoprotein, and low-density lipoprotein, alongside lower high-density lipoprotein levels (all p<0.05).

Conclusion

The study highlights a significant association between chronic plaque psoriasis and MS, reinforcing the need for early screening and integrated management of metabolic risk factors in psoriatic patients. A significant proportion of patients with chronic plaque psoriasis exhibit features of MS, even in the absence of clinically severe disease. These findings reinforce the need for routine MS screening in all psoriatic patients, regardless of severity scores (PASI or BSA). Dermatologists, often the first point of contact for these patients, can play a vital role in the early identification and management of metabolic risk factors through lifestyle modification and multidisciplinary care. Larger, multi-centric studies with control groups are recommended to confirm these associations and explore the pathophysiological mechanisms further.

## Introduction

Psoriasis is a common, chronic, and recurrent inflammatory skin disease, affecting approximately 2-3% of the global population. It presents with diverse cutaneous manifestations, most notably persistent, symmetrical, erythematous, scaling papules and plaques. Histopathologically, psoriasis is characterized by parakeratosis and epidermal hyperproliferation. Importantly, the disease is associated with long-term systemic inflammation, resulting from elevated pro-inflammatory cytokine production and continuous activation of both innate and adaptive immune pathways. This systemic inflammatory burden contributes to a range of comorbid conditions, including psoriatic arthritis, Crohn’s disease, depression, malignancies, cardiovascular diseases, and metabolic syndrome (MS) [1].

Among all comorbidities, MS is one of the most prevalent and clinically significant comorbidities linked to psoriasis [1]. Several studies have demonstrated associations between psoriasis and components of MS, such as obesity, hypertension, diabetes mellitus, dyslipidemia, and non-alcoholic fatty liver disease (NAFLD) [2-4]. Patients with severe forms of psoriasis are at a higher risk of developing MS compared to those with milder disease. The presence of MS also contributes to increased cardiovascular morbidity and reduced life expectancy in psoriatic individuals. Therefore, understanding the pathophysiological mechanisms connecting psoriasis and MS is of critical importance [1].

Epidemiological studies from the United States, Europe, and Japan have consistently reported a positive association between psoriasis and MS [2]. In India, the prevalence of psoriasis is estimated to range between 0.44% and 2.8%, with chronic plaque psoriasis being the most common clinical variant [5]. Furthermore, psoriasis has been identified as an independent risk factor for cardiovascular disease. This may be attributed to shared risk factors such as obesity, smoking, physical inactivity, and psychological stress, which are commonly observed in psoriatic patients [2].

Atherogenic dyslipidemia, defined by elevated levels of low-density lipoprotein (LDL), lipoprotein A, and total cholesterol, along with reduced levels of high-density lipoprotein (HDL) and apolipoprotein B, has also been frequently reported in individuals with psoriasis. Mallbris et al. observed that patients with newly diagnosed psoriasis (less than one year duration) exhibited significantly higher levels of LDL and apolipoprotein A-1, as well as altered cholesterol/triglyceride ratios, compared to healthy controls [6].

Standard biochemical screening tools, including fasting blood glucose and HbA1c, are essential, cost-effective methods for identifying metabolic disturbances in clinical settings [7,8]. Clinical scoring tools such as the Psoriasis Area and Severity Index (PASI) and Body Surface Area (BSA) are standardized, validated instruments used to objectively assess disease severity in psoriasis. These tools help quantify the extent and severity of cutaneous involvement, aiding in clinical evaluation, treatment planning, and monitoring of disease progression [9]. In light of the growing evidence linking psoriasis with MS, the present study was conducted to investigate this association in patients with histopathologically confirmed chronic plaque psoriasis.

## Materials and methods

Study design

This was a single-center, prospective, observational, descriptive, and hospital-based cross-sectional study conducted in the Department of Pathology at Smt. B. K. Shah Medical Institute and Research Centre, Vadodara, Gujarat, between January 2022 and June 2023. The study was conducted after obtaining ethical clearance from the Sumandeep Vidyapeeth Institutional Ethics Committee (approval number: SVIEC/284). MS was defined using the criteria established by the National Cholesterol Education Program Adult Treatment Panel III (NCEP-ATP III). According to these guidelines, MS is diagnosed when three or more of the following five criteria are met: (a) waist circumference >102 cm in men or >88 cm in women; (b) triglycerides ≥150 mg/dL; (c) HDL cholesterol <40 mg/dL in men or <50 mg/dL in women; (d) blood pressure ≥130/85 mmHg; (e) fasting blood glucose ≥110 mg/dL [10]. Patients with histopathologically confirmed chronic plaque psoriasis who presented for regular follow-up were enrolled. Patients with MS associated with other dermatological conditions such as acne vulgaris, hidradenitis suppurativa, androgenetic alopecia, acanthosis nigricans, or atopic dermatitis were excluded. Biopsy samples were obtained from all included cases for histopathological examination.

Tissue fixation and processing

Skin biopsy specimens were fixed in 10% neutral buffered formalin. A detailed gross examination was performed, and representative sections were selected from characteristic areas. Tissue samples underwent routine processing using a fully automated tissue processor (Leica TP1020; Leica Biosystems, Nussloch, Germany) through graded alcohols, xylene, and paraffin wax. Following processing, paraffin blocks were prepared. Sections of 3-5 μm thickness were cut using a rotary microtome (Leica RM2245; Leica Biosystems, Nussloch, Germany). For histological examination, sections were floated on a 45°C water bath and mounted on albumin-coated slides, then stained with hematoxylin-eosin (H&E). Special stains, such as Ziehl-Neelsen (ZN), were part of the routine protocol and were applied only when clinically indicated to rule out differential diagnoses. They were not required for the confirmation of psoriasis in this study.

Blood sample collection and biochemical analysis

Venous blood samples were collected after an overnight fast (minimum 12 hours). For fasting blood sugar, 1 mL of blood was collected in a sodium fluoride-containing vacutainer; for HbA1c, 2 mL was collected in an ethylenediaminetetraacetic acid (EDTA) vacutainer; and for lipid profile analysis, 2 mL was collected in a vacutainer containing powdered glass clot activator. All samples were processed within 2-4 hours of collection. Fasting blood glucose levels were analyzed using the Erba EM 200 and EM 300 fully automated chemistry analyzers (Erba Mannheim GmbH, Mannheim, Germany). HbA1c was measured using the HbA1c kit (Agappe Diagnostics Ltd., India). Lipid profile parameters were assessed using a fully automated hematology analyzer (Beckman Coulter AU480; Beckman Coulter Inc., California, USA).

PASI

The severity of psoriasis was assessed using PASI. This involved evaluating four anatomical regions separately: the head, upper limbs, trunk, and lower limbs. In each region, three clinical features - erythema, induration, and scaling - were scored on a scale from 0 to 4, with 0 representing no involvement, 1 as mild, 2 as moderate, 3 as severe, and 4 as very severe. The extent of area involvement for each site was also recorded and categorized into six grades: score 1 for involvement of ≤10% of the area, score 2 for 10-29%, score 3 for 30-49%, score 4 for 50-69%, score 5 for 70-89%, and score 6 for ≥90% involvement [11].

BSA 

The BSA involved in psoriasis was estimated using the commonly practiced "rule of nines," along with the patient’s palm method, where the palmar surface of the patient’s hand (including fingers) is considered equivalent to approximately 1% of their total BSA. Based on the extent of skin involvement, disease severity was categorized as follows: less than 5% BSA involvement was classified as mild, 5% to 10% as moderate, and greater than 10% as severe [12].

Statistical analysis

Statistical analysis was performed using IBM SPSS Statistics v25.0 (IBM Corp., Armonk, USA). Descriptive statistics were expressed as mean and SD for quantitative variables and as frequencies and percentages for qualitative variables. Comparison between quantitative variables was conducted using the unpaired t-test. For qualitative variables, comparisons between groups were made using the chi-square test or Fisher’s exact test, as appropriate. Categorical variables with 2×2 contingency tables were analyzed with Yates' continuity correction. A p-value of <0.05 was considered statistically significant.

## Results

**Figure 1:**
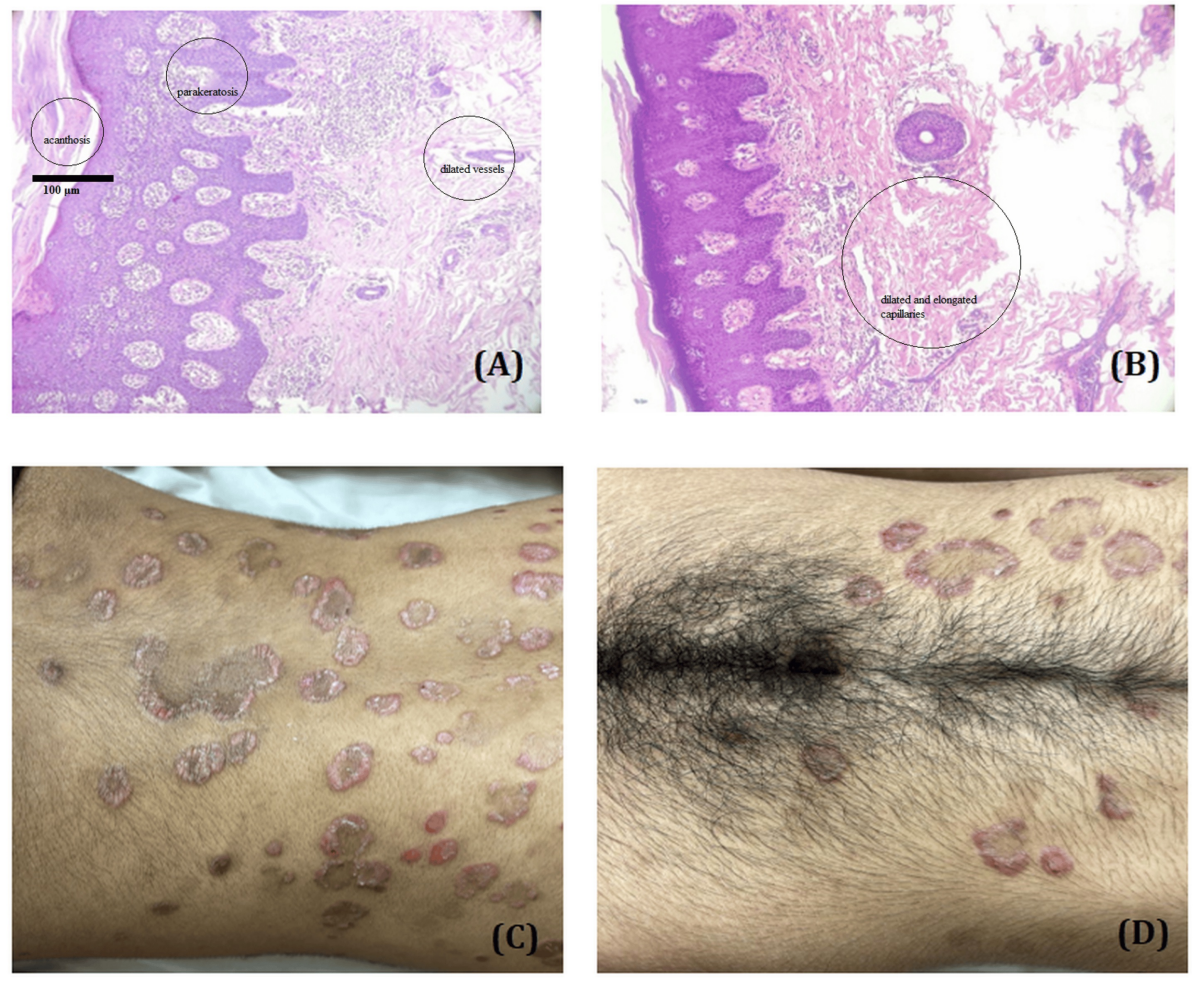
Representative clinical and histopathological features of chronic plaque psoriasis. (A) H&E-stained section (10× magnification) showing regular acanthosis with elongated rete ridges, hypogranulosis, and parakeratosis with neutrophilic aggregates (Munro’s microabscesses). (B) H&E-stained section (10× magnification) highlighting dilated and elongated capillaries in the dermal papillae and mounds of parakeratosis with mild epidermal hyperplasia. (C, D) Clinical images of chronic plaque psoriasis showing well-demarcated erythematous plaques with silvery white scaling distributed over the lower back and gluteal region. H&E: Hemotoxylin-eosin

MS was diagnosed based on the presence of three or more criteria as defined by the NCEP-ATP III. Among the 43 patients with psoriasis included in the study, six patients (14%) were found to have MS, while 37 patients (86%) did not meet the criteria for MS (Figure 1).

The mean age of patients without MS was 39.43 ± 13.43 years, while it was higher in those with MS (47.83 ± 21.78 years). The majority of patients in both groups were aged 31-50 years (19/37, 51.4% in non-MS; 2/6, 33.3% in MS). Male patients predominated in both groups (31/37, 83.8% in non-MS; 5/6, 83.3% in MS). A higher proportion of married individuals was observed among patients with MS (6/6, 100%) compared to non-MS (25/37, 67.6%). Most patients in the non-MS group were non-vegetarian (20/37, 54.1%), while a majority of MS patients were vegetarian (5/6, 83.3%). Socioeconomically, most patients in both groups belonged to the middle class (20/37, 54.1% in non-MS; 4/6, 66.7% in MS). A history of smoking was present in 14/37 (37.8%) non-MS patients and 3/6 (50.0%) MS patients, while alcohol consumption was noted in 13/37 (35.1%) non-MS patients and 2/6 (33.3%) MS patients. None of these differences were statistically significant (p>0.05 for all variables) (Table 1).

**Table 1:** Sociodemographic determinants of enrolled patients. MS: Metabolic syndrome

Variable	Domain	Non-MS (n=37)		MS (n=6)		P-value
		Number	Percent	Number	Percent	
Age group	Mean age	39.43 ± 13.43	-	47.83 ± 21.78	-	0.202
	18-30 years	12	32.4	2	33.3	0.561
	31-50 years	19	51.4	2	33.3	
	>50 years	6	16.2	2	33.3	
Gender	Male	31	83.8	5	83.3	0.668
	Female	6	16.2	1	16.7	
Marital status	Married	25	67.6	6	100	0.249
	Unmarried	12	32.4	0	0	
Food habits	Veg	17	45.9	5	83.3	0.207
	Non-veg	20	54.1	1	16.7	
Socioeconomic status	Lower class	17	45.9	2	33.3	0.564
	Middle class	20	54.1	4	66.7	
Addiction history	Smoking	14	37.8	3	50	0.908
	Alcohol	13	35.1	2	33.3	1.000

Nail changes (pitting, onycholysis, and subungual hyperkeratosis) were observed in 5/37 (13.5%) of patients without MS, while none of the MS patients exhibited nail involvement (0/6, 0%, p=0.786). In terms of disease severity, a greater proportion of non-MS patients had a PASI score of less than 5 (25/37, 67.6%) compared to MS patients (2/6, 33.3%). Conversely, a higher percentage of MS patients (4/6, 66.7%) had PASI scores between 5-6, though this difference was not statistically significant (p=0.245). When assessed for BSA involvement, mild disease (≤3% BSA) was more frequent in non-MS patients (28/37, 75.7%), while moderate disease (3-10% BSA) was significantly more common among MS patients (4/6, 66.7% vs 9/37, 24.3%, p=0.036) (Table 2).

**Table 2:** Clinical determinants of enrolled patients. *p<0.05, significant MS: Metabolic syndrome; PASI: Psoriasis Area and Severity Index; BSA: Body Surface Area

Variable	Domain	Non-MS (n=37)		MS (n=6)		P-value
		Number	Percent	Number	Percent	
Nail changes	Yes	5	13.5	0	0	0.786
PASI score	<5	25	67.6	2	33.3	0.245
	5-6	12	32.4	4	66.7	
BSA score	Mild (3%)	28	75.7	2	33.3	0.036*
	Moderate (3-10%)	9	24.3	4	66.7	

There was no statistically significant difference in mean height between psoriasis patients with and without MS. However, the mean weight was significantly higher in patients with MS (84.50 ± 16.44 kg) compared to those without MS (64.32 ± 11.47 kg). Similarly, the mean BMI was significantly elevated in the MS group (26.99 ± 5.48 kg/m^2^) versus the non-MS group (23.45 ± 2.84 kg/m^2^). The mean waist circumference was also significantly greater among patients with MS (94.50 ± 25.45 cm) than those without MS (74.54 ± 7.19 cm). In terms of blood pressure, both systolic (SBP) and diastolic (DBP) values were significantly higher in the MS group, with mean SBP of 140.33 ± 12.98 mmHg versus 122.59 ± 11.59 mmHg and mean DBP of 87.00 ± 5.62 mmHg versus 79.08 ± 4.72 mmHg in the non-MS group (Table 3).

**Table 3:** Anthropometrics and hemodynamics. *p<0.05, significant MS: Metabolic syndrome

Anthropometrics and hemodynamics	Non-MS		MS		P-value
	Mean	SD	Mean	SD	
Height (cm)	165.08	8.93	166.67	9.91	0.693
Weight (kg)	64.32	11.47	84.50	16.44	0.001*
BMI (kg/m^2^)	23.45	2.84	26.99	5.48	0.019*
Waist circumference (cm)	74.54	7.19	94.50	25.45	0.001*
Systolic blood pressure (mmHg)	122.59	11.59	140.33	12.98	0.001*
Diastolic blood pressure (mmHg)	79.08	4.72	87.00	5.62	0.001*

The mean random blood sugar levels were significantly higher in psoriasis patients with MS (123.67 ± 17.22 mg/dL) compared to those without MS (93.08 ± 13.91 mg/dL). Similarly, mean total cholesterol levels were significantly elevated in the MS group (243.83 ± 56.30 mg/dL) versus the non-MS group (172.89 ± 29.27 mg/dL). Mean triglyceride levels were also notably higher in patients with MS (233.33 ± 91.43 mg/dL) compared to those without MS (144.92 ± 62.12 mg/dL). In addition, the mean very low-density lipoprotein (VLDL) levels were significantly higher in the MS group (39.16 ± 16.41 mg/dL) than in the non-MS group (25.75 ± 10.72 mg/dL). Likewise, mean LDL levels were elevated in patients with MS (149.83 ± 47.91 mg/dL) compared to those without MS (104.00 ± 32.73 mg/dL). Conversely, mean HDL levels were significantly lower in psoriasis patients with MS (34.83 ± 9.04 mg/dL) than in those without MS (53.97 ± 16.48 mg/dL) (Table 4).

**Table 4:** Biochemical profile. *p<0.05, significant MS: Metabolic syndrome

Biochemical profile	Non-MS		MS		P-value
	Mean	SD	Mean	SD	
Random blood sugar (mg/dL)	93.08	13.91	123.67	17.22	0.001*
Cholesterol (mg/dL)	172.89	29.27	243.83	56.30	0.001*
Triglycerides (mg/dL)	144.92	62.12	233.33	91.43	0.004*
Very low-density lipoprotein (mg/dL)	25.75	10.72	39.16	16.41	0.012*
Low-density lipoprotein (mg/dL)	104.00	32.73	149.83	47.91	0.005*
High-density lipoprotein (mg/dL)	53.97	16.48	34.83	9.04	0.009*

## Discussion

MS was first described in 1988 by Stanford University endocrinologist Gerald Reaven [13]. He observed that the coexistence of four conditions - central obesity, dyslipidemia, hypertension, and glucose intolerance - in a single individual significantly increased the risk of cardiovascular disease. Since the 1950s, numerous studies have highlighted the association between psoriasis and various components of MS [2,6,14,15]. Psoriasis is a chronic, immune-mediated inflammatory skin disease affecting approximately 2-3% of the global population. Clinically, it presents with well-demarcated, erythematous, scaly plaques, typically located on extensor surfaces. Histopathologically, chronic plaque psoriasis is characterized by features such as parakeratosis, regular acanthosis, hypogranulosis, elongated rete ridges, dilated and tortuous capillaries in the dermal papillae, and perivascular lymphocytic infiltration. These hallmark features confirm diagnosis and help differentiate psoriasis from other dermatoses [16]. Patients with psoriasis are at an elevated risk of developing MS [17].

In the present study, among a total of 43 patients with psoriasis, six patients (14%) were diagnosed with MS. Multiple previous studies have established a strong association between psoriasis and MS. Using the NCEP-ATP III criteria, Gisondi et al. evaluated 338 patients with chronic plaque psoriasis and 334 controls, reporting a significantly higher prevalence of MS among psoriasis patients compared to controls [14]. Similarly, Zindancı et al. found a higher prevalence of MS in patients (53%) than in controls (39%) in their study involving 115 plaque-type psoriasis cases and 140 healthy individuals[17]. Nisa and Qazi, in a study of 150 patients with chronic plaque psoriasis and 150 healthy individuals, found MS in 28% of cases and 6% of controls [15]. According to the NCEP-ATP III criteria, Lakshmi et al. reported a prevalence of MS in 32.5% of psoriasis patients compared to 30% in controls [12]. Mebazaa et al. found a slightly higher MS prevalence among psoriasis patients (35.5%) than controls (30.8%) in their study involving 164 patients and 216 controls [3]. In contrast, Kim et al., in their study of 490 psoriasis patients and 682 controls, found no statistically significant difference in MS prevalence between the two groups [18].

In the current study, PASI scores were <5 in 25/37 (67.6%) and between 5-6 in 12/37 (32.4%) of psoriasis patients. Among patients with MS, 4/7 (66.7%) had a higher PASI score (5-6), although the difference was not statistically significant. Regarding BSA, 28/37 (75.7%) of patients had mild involvement, and 9/37 (24.3%) had moderate involvement. Among those with MS, 4/7 (66.7%) had moderate BSA involvement, indicating a significant association. However, Lakshmi et al. reported that MS was independent of PASI and BSA involvement in psoriasis [12]. Similar findings were reported by Nisa and Qazi as well as Gisondi et al., who found no correlation between MS prevalence and PASI or BSA scores [14,15]. Additionally, Zindancı et al. and Mebazaa et al. concluded that psoriasis severity (based on PASI) was not associated with MS prevalence [3,17]. However, Kim et al. observed a positive correlation between MS and severe psoriasis [18].

The mean weight was significantly higher in psoriasis patients with MS (84.50 ± 16.44 kg) compared to those without MS (64.32 ± 11.47 kg). Lakshmi et al. similarly reported mean weights of 56.35 kg in cases and 62.47 kg in controls [12]. The mean BMI was significantly higher in psoriasis patients with MS (26.99 ± 5.48 kg/m^2^) compared to those without MS (23.45 ± 2.84 kg/m^2^). Lakshmi et al. reported BMI values of 22.47 in cases and 21.53 in controls [12]; Gisondi et al. found BMI values of 27.7 in cases and 25.4 in controls [14]; Nisa and Qazi reported BMI values of 23.94 ± 3.66 in cases and 25.4 ± 4.9 in controls [15]; and Ferdinando et al. noted BMI values of 28.4 ± 5.09 in cases and 26.8 ± 4.68 in controls [19].

Waist circumference was significantly greater in psoriasis patients with MS (94.50 ± 25.45 cm) than in those without MS (74.54 ± 7.19 cm). Similar findings were reported by Lakshmi et al., who observed waist circumferences of 88.27 cm in cases and 86.33 cm in controls [12], while Ferdinando et al. reported medians of 99 cm in cases and 94 cm in controls [19].

SBP was significantly higher in psoriasis patients with MS (140.33 ± 12.98 mmHg) compared to those without MS (122.59 ± 11.59 mmHg). Lakshmi et al. reported mean SBP values of 121.8 mmHg in cases and 118.67 mmHg in controls [12]. Ferdinando et al. reported median SBP values of 130 mmHg in cases and 120 mmHg in controls [19], while Kothiwala et al. observed mean SBP values of 129.4 ± 14.42 mmHg in cases and 121.5 ± 11.90 mmHg in controls [20].

DBP was also significantly higher in psoriasis patients with MS (87.00 ± 5.62 mmHg) versus those without MS (79.08 ± 4.72 mmHg). Lakshmi et al. reported DBP values of 78.9 mmHg in cases and 77.56 mmHg in controls [12], while Ferdinando et al. observed a median DBP of 80 mmHg in both groups [19], and Kothiwala et al. reported mean values of 82.3 ± 9.30 mmHg in cases and 77.8 ± 9.14 mmHg in controls [20].

The mean random blood sugar levels were significantly higher in psoriasis patients with MS (123.67 ± 17.22 mg/dL) compared to those without MS (93.08 ± 13.91 mg/dL). Ferdinando et al. reported fasting blood sugar levels of 89 mg/dL in cases and 91.7 mg/dL in controls [19], and Kothiwala et al. noted mean fasting values of 98.5 ± 16.82 mg/dL in cases and 91.1 ± 12.82 mg/dL in controls [20].

Total cholesterol levels were significantly elevated in psoriasis patients with MS (243.83 ± 56.30 mg/dL) compared to those without MS (172.89 ± 29.27 mg/dL). Lakshmi et al. reported cholesterol levels of 171.7 mg/dL in cases and 174.07 mg/dL in controls [12], while Ferdinando et al. observed medians of 181 mg/dL in cases and 180 mg/dL in controls [19].

Triglyceride levels were also significantly higher in psoriasis patients with MS (233.33 ± 91.43 mg/dL) than in those without MS (144.92 ± 62.12 mg/dL). Lakshmi et al. observed mean triglycerides of 149.5 mg/dL in cases and 137.44 mg/dL in controls [12], while Ferdinando et al. reported medians of 118 mg/dL in cases and 119.2 mg/dL in controls [19], and Kothiwala et al. noted means of 130.2 ± 69.83 mg/dL in cases and 142.3 ± 102.88 mg/dL in controls [20].

VLDL levels were significantly elevated in psoriasis patients with MS (39.16 ± 16.41 mg/dL) compared to those without MS (25.75 ± 10.72 mg/dL). Lakshmi et al. reported VLDL levels of 25.45 mg/dL in cases and 23.92 mg/dL in controls [12]. LDL was also significantly higher in patients with MS (149.83 ± 47.91 mg/dL) compared to those without MS (104.00 ± 32.73 mg/dL). Lakshmi et al. reported LDL levels of 109.21 mg/dL in cases and 111.96 mg/dL in controls [12], while Ferdinando et al. observed median LDL values of 196.4 mg/dL in cases and 104 mg/dL in controls [19].

Conversely, HDL was significantly lower in psoriasis patients with MS (34.83 ± 9.04 mg/dL) than in those without MS (53.97 ± 16.48 mg/dL). Lakshmi et al. found mean HDL levels of 37.7 mg/dL in cases and 38.81 mg/dL in controls [12], while Kothiwala et al. reported means of 41.1 ± 7.73 mg/dL in cases and 40.9 ± 4.45 mg/dL in controls [20].

There are shared immunological mechanisms between psoriasis and MS. Intra-abdominal fat, functioning as an endocrine organ, releases adipocytokines that influence vascular endothelial function, glucose metabolism, and inflammatory pathways [4]. Increased levels of IL-6, PAI-1, and TNF-α, commonly associated with visceral obesity, have also been found in psoriasis [21,22]. Leptin, another adipocyte-derived hormone, modulates cytokine production and affects both type 1 T-helper (Th1) and type 2 T-helper (Th2) responses, contributing to acute and chronic inflammation. Hyperleptinemia has been implicated in MS, and elevated leptin levels have also been reported in psoriasis, though their precise role remains unclear [23].

This study has several strengths. Firstly, all cases of psoriasis included were confirmed histopathologically, ensuring diagnostic accuracy. Secondly, the study employed standardized scoring systems such as PASI and BSA for objective assessment of disease severity. Thirdly, detailed clinical, biochemical, and anthropometric data were collected, allowing for a comprehensive evaluation of metabolic syndrome components. Finally, this is one of the few studies from western India exploring the clinicopathological association between psoriasis and MS, contributing valuable regional data to the existing literature. This study has certain limitations. Firstly, the small sample size limited the number of patients diagnosed with MS. Secondly, the absence of a control group restricts comparative analysis. Finally, as a single-center study, the findings cannot be generalized to the broader population.

Based on the observed association between chronic plaque psoriasis and MS, we recommend that all patients diagnosed with psoriasis, regardless of disease severity, should undergo routine screening for components of MS, including blood pressure, waist circumference, lipid profile, and fasting blood glucose or HbA1c. Dermatologists should collaborate closely with internists or endocrinologists for the early identification and management of metabolic risk factors. Patients with moderate BSA involvement, even in the absence of high PASI scores, should be considered at higher risk of MS and monitored accordingly. Patient education on lifestyle modifications such as healthy diet, physical activity, weight management, and smoking/alcohol cessation should be an integral part of psoriasis management. These recommendations aim to facilitate early detection and intervention for MS, thereby reducing long-term cardiovascular risk in patients with psoriasis.

Future studies with larger sample sizes and control groups are warranted to better establish the association between MS and psoriasis. We agree that interventional studies, particularly those incorporating mind-body interventions such as yoga, meditation, and stress-reduction techniques, represent a promising area of research in the management of psoriasis. These approaches could potentially modulate systemic inflammation and influence disease activity. Although our current study was observational and cross-sectional in nature, we acknowledge the value of longitudinal studies that assess changes in clinical severity scores such as PASI and BSA over time in response to structured interventions. Additionally, exploring the dose-dependent effects of such non-pharmacologic therapies could provide important evidence for integrated, holistic management of psoriasis and its comorbidities, including metabolic syndrome.

## Conclusions

Dermatologists, often being the first point of contact for patients with psoriasis, play a crucial role in identifying and addressing underlying metabolic abnormalities. The findings of the present study underscore the importance of screening all psoriasis patients for MS and concurrently providing education on lifestyle modifications. Such a comprehensive approach is particularly vital when initiating systemic therapies, ensuring a more holistic management of these patients. However, to elucidate the underlying mechanisms of this association and to assess the impact of systemic treatments on metabolic parameters, further large-scale and well-designed studies are warranted.
